# Screening for High-Risk Oral Human Papillomavirus (HPV31, HPV33, HPV35) in a Multi-Racial Pediatric and Adult Clinic Patient Population

**DOI:** 10.3390/cancers15184501

**Published:** 2023-09-10

**Authors:** Hunter Hinton, Spencer Coleman, J. R. Salem, Karl Kingsley

**Affiliations:** 1 Department of Advanced Education in Orthodontics, School of Dental Medicine, University of Nevada-Las Vegas, 1700 W. Charleston Boulevard, Las Vegas, NV 89106, USA; hintoh2@unlv.nevada.edu; 2 Department of Clinical Sciences, School of Dental Medicine, University of Nevada-Las Vegas, 1700 W. Charleston Boulevard, Las Vegas, NV 89106, USA; colems6@unlv.nevada.edu (S.C.); salemw1@unlv.nevada.edu (J.R.S.); 3 Department of Biomedical Sciences, School of Dental Medicine, University of Nevada-Las Vegas, 1001 Shadow Lane Boulevard, Las Vegas, NV 89106, USA

**Keywords:** high-risk HPV, human papillomavirus (HPV), oral cancer screening, qPCR screening

## Abstract

**Simple Summary:**

Many previous studies have focused on the prevalence of high-risk HPV16 and HPV18 strains, highly associated with cervical and oral cancers covered by the original HPV vaccine. However, little research is currently available regarding the oral prevalence of other high-risk HPV strains, such as HPV31 and HPV33, which are part of the recently revised nine-strain HPV vaccine. This study conducted one of the first oral prevalence studies of these high-risk HPV strains among a multi-ethnic patient population at a public dental school in Nevada. The results of this investigation revealed a significant percentage of children and adults in this study harbored one or more of these high-risk strains, which were mostly found among patients within the recommended vaccination or catch-up age range (9–45 years).

**Abstract:**

Many human papillomavirus (HPV) strains induce cancer in the cervix and the oral cavity. Although high-risk strains including HPV16 and HPV18 are commonly known, additional high-risk strains including HPV31, HPV33, and HPV35 may also induce carcinogenesis, and much less is known about their prevalence. Using an approved protocol, samples from a salivary biorepository were screened to find pediatric and adult samples from a multi-ethnic, university-based patient clinic population. A total of *N* = 86 samples from the saliva biorepository met the quality and concentration standards and were screened for high-risk HPV. qPCR screening of adult samples revealed *n* = 10/45 or 22% were HPV31- or HPV33-positive. In addition, a total of *n* = 9/41 or 21.9% of pediatric samples were either HPV31- or HPV33-positive (or both). No samples harbored HPV35. Most samples were derived from patients within the recommended vaccination or catch-up age range (age 9–45 years). These results demonstrated that a significant percentage of patients harbor additional high-risk HPV strains within the oral cavity, including HPV31 and HPV33. These data support oral healthcare provider recommendations for the newer nine-valent vaccine, which includes both HPV31 and HPV33.

## 1. Introduction

Human papillomavirus (HPV) is a non-enveloped, double-stranded DNA virus that is epitheliotropic in nature and is known to cause disease in a variety of tissues [1,2]. There are between 150–200 different strains of HPV, some of which are known to cause human disease, including cancer [3,4]. Approximately 40 types of HPV are known to cause cutaneous or mucosal infections within human hosts [5,6].

HPV infections can spread through skin-to-skin contact through both sexual and non-sexual transmission pathways [7,8]. Epidemiologic prevalence estimates suggest that more than 40 million people in the United States are currently infected with some form of HPV, with an incidence of more than 10 million new cases per year [9,10,11]. Although most HPV infections are cleared by the immune system (90–95% by some estimates), some infections result in long-term infections that cause precancerous abnormalities and malignancies if left untreated [12,13,14].

The main clinical distinction between the many human papillomavirus (HPV) strains lies in their ability to mediate cellular transformation into cancer within various tissues, which are broadly categorized into low-risk (LR) or high-risk (HR) depending upon their most frequently associated clinical outcomes [15,16]. The low-risk HPV strains 6 and 11 are responsible for >90% of anogenital warts and lesions, but also include additional commonly identified strains such as 40, 42, 43, and 44 [17,18]. In addition, high-risk strains HPV16 and 18 are responsible for the vast majority of cervical as well as the majority of HPV-associated oropharyngeal cancers, although there are many additional high-risk HPV strains commonly identified including HPV31, 33, 35, 39, 45, 51, 52, 56, 58, 59, 66, and 68 [19,20].

Although the high-risk strains HPV16 and HPV18 are the most frequently identified, additional high-risk strains found in the cervix and oral cavity may induce carcinogenesis, including HPV31, HPV33, and HPV35, although much less is known about their prevalence [21,22,23]. Based upon this understanding of additional clinically-relevant strains of HPV, the new nine-valent HPV vaccine incorporates not only the most common high-risk (16 and 18) and low-risk (6 and 11) HPV strains, but also includes additional high-risk strains such as HPV31, 33, 45, 52, and 58 [24,25,26]. Although several studies from this institution have evaluated the prevalence of high-risk oral HPV strains 16 and 18 among both adult and pediatric patient populations, no study to date has evaluated the additional high-risk strains of HPV31, 33, and 35 [27,28,29,30].

Based upon lack of evidence regarding these high-risk strains, the primary objective of this project is to evaluate the prevalence of these strains among pediatric and adult patients through screening of clinical saliva samples. The working hypothesis for this project was that additional high-risk strains of HPV, such as HPV31, 33, and 35, would be identified if the appropriate screening for these additional HPV strains was conducted.

## 2. Materials and Methods

### 2.1. Study Approval

The review of the protocol for this study was completed by the Office for the Protection of Research Subjects (OPRS) and the Institutional Review Board at the University of Nevada, Las Vegas (UNLV). Protocol #1717625-1, titled “Retrospective analysis of microbial prevalence from DNA isolated from saliva samples originally obtained from the University of Nevada, Las Vegas (UNLV) School of Dental Medicine (SDM) pediatric and clinical population”, was approved by the UNLV-IRB and OPRS on 3 March 2021.

### 2.2. Human Subjects and Informed Consent

The original collection protocol was approved by the UNLV-IRB under protocol OPRS#1305-4466M, “The Prevalence of Oral Microbes in Saliva from the UNLV School of Dental Medicine Pediatric and Adult Clinical Population”. Inclusion criteria included patients between the ages of 5 to 45 years of age that agreed to provide informed consent (adult over 18 years of age) or pediatric assent with Informed consent (children under 18 years of age with guardian or parental permission and consent). Exclusion criteria included any patients (or parents/guardians that refused to provide informed consent or pediatric assent) and any samples from patients outside the UNLV School of Dental Medicine.

### 2.3. Original Sample Collection Protocol

Original sample collection from UNLV–SDM clinic patients involved only voluntary study participants. Following voluntary agreement to study participation and the provision of informed consent and/or pediatric assent, clinic patients were provided a sterile 50 mL saliva collection tube. Up to 5.0 mL of unstimulated saliva was collected from each study participant. To prevent the collection of any personal or patient information, all samples were assigned a non-duplicated, randomly-generated number for labeling purposes. Only basic demographic characteristics, such as patient age at the time of collection, self-reported ethnicity or race, and sex were noted at the time of sample collection. All samples and other materials were stored in a biomedical laboratory for subsequent analysis and processing. 

### 2.4. DNA Isolation and Analysis

A total of N = 253 samples from the biomedical sample repository were identified for potential inclusion in the current retrospective analysis. DNA was isolated from each sample using the phenol:chloroform extraction method. In brief, samples were thawed and vortexed, then 500 µL was transferred to a sterile microcentrifuge tube and mixed with 500 µL of TRIzol DNA isolation reagent from Invitrogen (Waltham, MA, USA). To each sample, 200 µL of molecular-grade chloroform from Invitrogen (Waltham, MA, USA) was added prior to incubation on ice for 15 min. Each sample was subsequently centrifuged using an Eppendorf Model 5425 (Hamburg, Germany) refrigerated microcentrifuge for fifteen minutes at 12,000 × relative centrifugal force (RCF) at 4 °C. 

The upper aqueous phase (approximately 400–500 µL) was transferred to a new, sterile microcentrifuge tube and mixed with molecular-grade isopropanol from Invitrogen (Waltham, MA, USA) to precipitate the DNA. Each sample was then centrifuged using the settings described above. Following removal of the isopropanol, the pellet containing sample DNA was washed using molecular-grade ethanol from Invitrogen (Waltham, MA, USA) prior to centrifugation for ten additional minutes. After removal of the ethanol supernatant, DNA from each sample was resuspended in 100 µL of nuclease-free water obtained from Thermo Fisher Scientific (Waltham, MA, USA). Analysis of quality and quantity of DNA was facilitated with a NanoDrop 2000 spectrophotometer obtained from Thermo Fisher Scientific (Waltham, MA, USA) using absorbances of A260 and A280 nm, as specified by the manufacturer protocol. All samples that met minimum quantity standards (>10 ng/µL) as well as quality standards (A260:A280 ratio > 1.65) were then utilized in the current study for molecular HPV screening using qPCR.

### 2.5. qPCR Screening

The samples that met the minimum criteria for DNA quantity and DNA purity (N = 86) were screened for high-risk HPV strains 31, 33, and 35 using quantitative polymerase chain reaction (qPCR). Each reaction consisted of 15 µL Fast SYBR Green Master Mix from Applied Biosystems (Waltham, MA, USA), 1.5 µL of forward primer, 1.5 µL of reverse primer, 2.0 µL of sample DNA, and 5.0 µL of nuclease-free water. Reactions were performed using the QuantStudio 3 from Thermo Fisher Scientific (Waltham, MA, USA) and the following validated primers:

HPV31 forward: ATTCCACAACATAGGAGGAAGGTG;
HPV31 reverse: CACTTGGGTTTCAGTACGAGGTCT;
HPV33 forward: ATATTTCGGGGTCGTTGGGCA;
HPV33 reverse: ACGTCACAGTGCAGTTTCTCTACGT;
HPV35 forward: TCGGTGTATGTCTGTTGGAAAC;
HPV35 reverse: CATAGTCTTGCAATGTAGTTATTTCTCCA.

### 2.6. Statistical Analysis

Demographic variables for the study sample were compiled and presented as simple, descriptive statistics. Analysis of differences between the study sample and the overall clinic population with respect to categorical variables, such as sex and race or ethnicity, were done using chi-square statistics which is appropriate for non-parametric data analysis. Analysis of qPCR screening results was also presented as simple descriptive statistics, such as percentages, and the differences between HPV-positive and HPV-negative samples were also analyzed using chi-square statistics and the GraphPad Prism software, Version 8 (San Diego, CA, USA). Comparisons for parametric data, such as age, were completed using two-tailed Student’s *t*-tests, using an alpha level of 0.05 for statistical significance.

## 3. Results

A total of *N* = 86 samples from an existing biorepository met the minimum DNA quality and quantity standards for inclusion in this retrospective study, which were nearly equally divided between adults (52.3% or n = 45/86, Table 1) and pediatric (47.7% or *n* = 41/86) patients (Table 2). Analysis of the adults in the study sample revealed approximately half were derived from females (55.6% or *n* = 25/45), which closely approximates the percentage of females in the overall clinic population (49.1%), *p* = 0.1614 (Table 1). In addition, analysis of the demographic characteristics demonstrated that the majority of the adult study samples were derived from racial or ethnic minorities (55.6% or *n* = 25/45), which was slightly lower but not significantly different from the percentages observed within the overall main clinic population (65.4%), *p* = 0.0592. Finally, the average age of the adult samples was found to be 41.5 years (range: 18 to 73 years), which closely approximates the average age of the main patient clinic population of 42.3 years (range: 18 to 89 years), *p* = 0.7738.

Analysis of the pediatric patients in the study sample revealed approximately half were derived from females (56.1% or *n* = 23/41), which was similar to the proportion of females observed from the pediatric clinic patient population (52.8%), *p* = 0.5478 (Table 2). Demographic analysis of the ethnic and racial characteristics of the study sample demonstrated that most patient samples from the pediatric clinic had self-identified as an ethnic or racial (non-White) minority (82.96% or *n* = 34/41), which was not deemed to be statistically significant from the observed proportion of minority (non-White) patients from the overall the pediatric clinic population (75.34%), *p* = 0.0647. In addition, the average age of samples within this study was 12.7 years, ranging between 5 and 17 years, which was slightly higher than the average age of the overall clinical population of pediatric patients of 10.4 years, ranging between 0 and 17 years, *p* = 0.2531.

**Figure 1:**
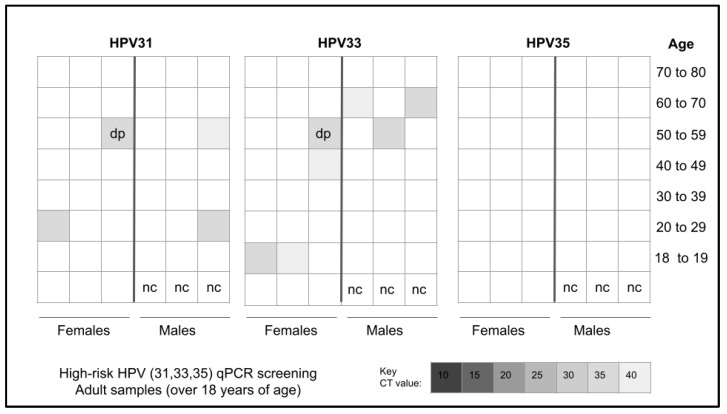
Heatmap analysis for qPCR screening of adult samples for high-risk HPV. A total of *n* = 10 samples tested positive for HPV with *n* = 4/10 or 40% testing positive for HPV31, *n* = 6/10 testing positive for HPV33, and one sample testing positive for both HPV31 and HPV33. No samples tested positive for HPV35. nc = negative control, dp = double positive.

Screening of the adult patients revealed that *n* = 10/45 or 22.2% different samples harbored one or more of the three high-risk strains of HPV analyzed, including 31, 33, and 35 (Figure 1). More specifically, *n* = 4/10 or 40% of the HPV-positive samples harbored HPV31 while *n* = 7/10 or 70% harbored HPV33, including one that was also positive for HPV31. However, none of the samples evaluated harbored HPV35.

More detailed analysis of the adult samples revealed an equal distribution of HPV-positive samples between males (*n* = 5/10 or 50%) and females (*n* = 5/10 or 50%), which closely matched the distribution of HPV-negative samples from males and females, *p* = 0.5478 (Table 3). In addition, analysis of race and ethnicity revealed the majority of HPV-positive samples were derived from minority patients (60%), which closely matched the proportion of HPV-negative samples from minority patients (54.3%), *p* = 0.2286. Finally, the proportion of HPV-positive samples and HPV-negative samples from patients within the catch-up range (under 45 years) was nearly equal and not significantly different (40%, 42.9%, respectively), *p* = 0.5445.

**Figure 2:**
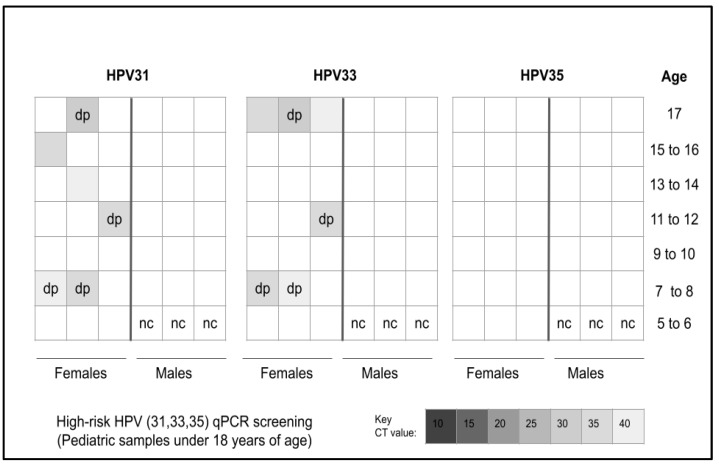
Analysis of heatmap for qPCR screening of pediatric samples for high-risk HPV. A total of *n* = 9/41 or 21.9% of samples tested positive for HPV with *n* = 6/9 or 66.7% testing positive for HPV31, *n* = 7/9 or 77.8% testing positive for HPV33 and four samples testing positive for both HPV31 and HPV33. No samples tested positive for HPV35. nc = negative control, dp = double positive.

Screening of the pediatric patient samples revealed that *n* = 9/41 or 21.9% samples harbored one or more of the three high-risk strains of HPV, such as 31, 33, and 35 (Figure 2). More specifically, *n* = 6/9 or 66.7% of the HPV-positive samples harbored HPV31 while *n* = 7/9 or 77.8% harbored HPV33—including four that were double positive for both HPV31 and HPV33. However, none of the samples evaluated harbored HPV35.

More detailed analysis of the pediatric samples revealed an unequal distribution of HPV-positive samples between males (*n* = 3/9 or 33.3%) and females (*n* = 6/9 or 66.7%), which was significantly different from the distribution of HPV-negative samples from males and females, *p* = 0.005 (Table 4). In addition, analysis of race and ethnicity revealed the majority of HPV-positive samples were derived from minority patients (*n* = 7/9 or 77.8%), which did not differ significantly from the proportion of HPV-negative samples from minority patients (*n* = 27/32 or 84.4%), *p* = 0.1017. Finally, the proportion of HPV-positive samples and HPV-negative samples from patients within the HPV vaccination age range (11 to 17 years) was significantly different (77.8%, 83.7%, respectively), *p* = 0.0001.

## 4. Discussion

The primary goal of this study was to assess the prevalence of high-risk HPV strains 31, 33, and 35 using an existing biorepository including both pediatric and adult clinical saliva samples. The results of this study successfully demonstrated that HPV31 and HPV33 were found among both pediatric and adult samples in similar proportions (21.9% and 22.2%, respectively), although no samples tested positive for HPV35. These data represent the first clinical descriptions of non-HPV16 and non-HPV18 high-risk HPV prevalence within this patient population [27,28,29,30].

However, some notable differences were found regarding the prevalence of these high-risk HPV strains compared with previous studies of HPV strains HPV16 and HPV18. For example, this study found nearly one-quarter of adults (22.2%) harbored either HPV31, HPV33, or both. This is somewhat lower than the most recent study of HPV16 and HPV18 prevalence among adults within this patient population, which found an overall prevalence of 30.2% [27]. However, it is also much higher than the first description of HPV16 and HPV18 prevalence among adults from nearly a decade earlier that found an overall prevalence of only 2.6% within the same clinical patient population [30]. Moreover, these data also confirm other recent observations of HPV31 and HPV33 oral prevalence within other patient populations, which ranged between 5.7% and 14.3% [31,32,33]. These data may therefore support the overwhelming evidence that HPV16 and HPV18 remain the dominant oral strains of concern, but that other high-risk HPV strains may also be present if the appropriate screening is performed.

In addition, these data also demonstrated that HPV31 and HPV35 were found in approximately one-fifth of pediatric patient samples (21.9%), which corresponds to similar prevalence levels of HPV16 and HPV18 found within these patients as recently as last year (19.5%) [27]. Although this represents the first non-HPV16, non-HPV18 screening within this patient population, the more troubling aspect is the rise in pediatric oral HPV prevalence observed with HPV16 and HPV18, which was 2.5% in 2012, 9.2% in 2016, and 19.5% in 2022 [27,28,29]. As more studies confirm oral prevalence levels of high-risk HPV among pediatric populations at similar levels, the case for screening and evaluating these additional HPV strains becomes more critical [34,35].

Despite the significance of epidemiological data regarding high-risk HPV prevalence, as more studies are now screening for HPV strains other than HPV16 and HPV18, there are some limitations associated with this type of study that should also be considered [36,37]. Specifically, this was a retrospective study of previously collected saliva samples from an existing biorepository and may not reflect the most current oral prevalence, which may have shifted due to behavioral and vaccination practice changes following the onset of the SARS-CoV-2 (COVID-19) pandemic [38,39]. In addition, due to the retrospective nature of this study, no other health information (oral or systemic) was available to determine if other factors, such as smoking, vaping, or oral microbiota, might have influenced the outcomes of this study [40,41,42]. Finally, due to the parameters of the original protocol, these samples were part of cross-sectional studies involving one-time saliva collections, and therefore have no information regarding the temporal nature of the HPV detected and whether each was a short- or long-term infection.

However, due to the fact that high-risk oral HPV strains may have important functions in both the development and progression of oral cancers, any information regarding the prevalence of these non-HPV16 and non-HPV18 strains becomes critical for oral health researchers, clinicians, and epidemiologists to evaluate and consider [43,44]. Moreover, the findings from this study that most of the HPV-positive samples were from vaccination age-appropriate patients also suggest that more emphasis on the public health benefits and prevention of HPV-related diseases through vaccination may have the potential to yield significant results through more widespread awareness of the risks and prevalence of high-risk HPV and acceptance of these effective and low-cost prevention methods [45,46].

## 5. Conclusions

The importance of these findings, including the increased prevalence of high-risk strains HPV31 and HPV33 detected in this study combined with previous data about the increasing prevalence of HPV16 and HPV18 within this patient population, may suggest a more robust and focused effort on both HPV vaccination and awareness of oral HPV infection [27,28,29,30]. The results further highlight this need since the oral prevalence of HPV31 and HPV33 previously published may be underreported with current screenings [31,32,33]. However, recent evidence regarding increasing levels of vaccine hesitancy also suggest that more evidence may be needed to demonstrate the relevance of HPV prevention, particularly among this patient population [47,48]. This study is among the first to provide this type of evidence through the assessment and evaluation of oral HPV infection outside of the conventional HPV strains of HPV16 and HPV18.

## Figures and Tables

**Table 1:** Demographic analysis of adult study samples.

Demographics	Study Sample Adults (*n* = 45)	UNLV–SDM Adult Clinic (N = 71,660)	Statistical Analysis
**Sex**			
Adult—Females	55.60% (*n* = 25/45)	49.10% (*n* = 35,185/71,660)	X^2^ = 1.961, d.f. = 1
Adult—Males	44.40% (*n* = 20/45)	50.90% (*n* = 36,475/71,660)	*p* = 0.1614
**Race**			
White	44.40% (*n* = 20/45)	34.60% (*n* = 24,794/71,660)	X^2^ = 3.560, d.f. = 1
Minority	55.60% (*n* = 25/45)	65.40% (*n* = 46,866/71,660)	*p* = 0.0592
Hispanic	28.80% (*n* = 13/45)	58.60% (*n* = 41,993/71,660)	
Black	13.30% (*n* = 6/45)	10.20% (*n* = 7309/71,660)	
Asian/Other	13.30% (*n* = 6/45)	6.60% (*n* = 4730/71,660)	
**Age**			
Mean	41.5 years	42.3 years	Two-tailed *t*-test
Range	18–73 years	18–89 years	*p* = 0.7738

**Table 2:** Demographic analysis of pediatric study samples.

Demographic	Study Sample Pediatrics (*n* = 41)	UNLV–SDM Pediatric Clinic (N = 24,849)	Statistical Analysis
**Sex**			
Pediatric—Females	56.10% (*n* = 23/41)	52.80% (*n* = 13,120/24,849)	X^2^ = 0.361, d.f. = 1
Pediatric—Males	43.90% (*n* = 18/41)	47.20% (*n* = 11,729/24,849)	*p* = 0.5478
**Race**			
White	17.10% (*n* = 7/41)	24.70% (*n* = 6138/24,849)	X^2^ = 3.413, d.f. = 1
Minority	82.90% (*n* = 34/41)	75.30% (*n* = 18,711/24,849)	*p* = 0.0647
Hispanic	56.10% (*n* = 23/41)	52.10% (*n* = 12,946/24,849)	
Black	9.80% (*n* = 4/41)	11.80% (*n* = 2932/24,849)	
Asian/Other	12.20% (*n* = 5/41)	11.40% (*n* = 2833/24,849)	
**Age**			
Mean	12.7 years	10.4 years	Two-tailed *t*-test
Range	5–17 years	0–17 years	*p* = 0.2531

**Table 3:** Demographic analysis of adult HPV-positive and HPV-negative samples.

Demographics	HPV-Positive (*n* = 10/45)	HPV-Negative (*n* = 35/45)	Statistical Analysis
Adult—Males	50% (*n* = 5/10)	42.9% (*n* = 15/35)	X^2^ = 0.361, d.f. = 1
Adult—Females	50% (*n* = 5/10)	57.1% (*n* = 20/35)	*p* = 0.5478
Total	22.2% (*n* = 10/45)	77.8% (*n* = 35/45)	
Adult—Non-Minority	40% (*n* = 4/10)	45.7% (*n* = 16/35)	X^2^ = 1.449, d.f. = 1
Adult—Minority	60% (*n* = 6/10)	54.3% (*n* = 19/35)	*p* = 0.2286
Total	22.2% (*n* = 10/45)	77.8% (*n* = 35/45)	
Below Catch-up Age (Under 45 years)	40% (*n* = 4/10)	42.9% (*n* = 15/35)	X^2^ = 0.367, d.f. = 1
Above Catch-up Age (Over 45 years)	60% (*n* = 6/10)	57.1% (*n* = 20/35)	*p* = 0.5445
Average Age	44.8 years	46.1 years	

**Table 4:** Demographic analysis of pediatric HPV-positive and HPV-negative samples.

Demographics	HPV-Positive (*n* = 9/41)	HPV-Negative (*n* = 32/41)	Statistical Analysis
Pediatric—Males	33.3% (*n* = 3/9)	56.9% (*n* = 15/32)	X^2^ = 7.868, d.f. = 1
Pediatric—Females	66.7% (*n* = 6/9)	53.1% (*n* = 17/32)	*p* = 0.005
Total	21.9% (*n* = 9/41)	78.1% (*n* = 32/41)	
Pediatric—Non-Minority	22.2% (*n* = 2/9)	15.6% (*n* = 5/32)	X^2^ = 2.679, d.f. = 1
Pediatric—Minority	77.8% (*n* = 7/9)	84.4% (*n* = 27/32)	*p* = 0.1017
Total	21.9% (*n* = 9/41)	78.1% (*n* = 32/41)	
Within Vaccination Age Range (11 to 17 years)	77.8% (*n* = 7/9)	93.7% (*n* = 30/32)	X^2^ = 45.390, d.f. = 1
Below Vaccination Age Range (Under 11 years)	22.2% (*n* = 2/9)	6.3% (*n* = 2/32)	*p* = 0.0001
Average Age	13.4 years	12.7 years	

## Data Availability

The data presented in this study are available on request from the corresponding author.
